# Patients’ Perceptions of Endodontic Treatment as Part of Public Health Services: A Qualitative Study

**DOI:** 10.3390/ijerph13050450

**Published:** 2016-04-27

**Authors:** José Leonardo Barbosa Melgaço-Costa, Renata Castro Martins, Efigênia Ferreira Ferreira, Antônio Paulino Ribeiro Sobrinho

**Affiliations:** 1 Departament of Restorative Dentistry, Faculty of Dentistry, Universidade Federal de Minas Gerais (UFMG), Belo Horizonte, Minas Gerais 31270 901, Brazil; leomelgaco@yahoo.com.br; 2 Departament of Social and Preventive Dentistry, Faculty of Dentistry, Universidade Federal de Minas Gerais (UFMG), Belo Horizonte, Minas Gerais 31270 901, Brazil; r.c.martins@uol.com.br (R.C.M.); efigeniaf@gmail.com (E.F.F.)

**Keywords:** health care quality, access and evaluation, patient satisfaction, secondary care, endodontic

## Abstract

Evaluations by patients constitute an important part of the process of improving health services. This study examined patients’ perceptions of secondary dental care in three cities in Minas Gerais, Brazil based on the endodontic treatment received. Data were collected using semi-structured interviews (addressing access, treatment and results) and a field diary (direct observations and report of professionals). The interviews were audiotaped, fully transcribed, and analyzed using content analysis. Two principal themes were identified: access to service and quality of service. The difficulties in accessing service were associated with the insufficient number of professionals to meet the high demand for endodontic treatment, problems in referring from primary to secondary care and geographic barriers. Service quality was related to the presence/absence of pain and anxiety that patients experienced, the time and number of sessions required to complete treatment, how patients were treated by dentists, and whether those patients would recommend the service to other patients. Access to endodontic treatment was a problem emphasized by users, and satisfaction with the quality of the service was more related to how patients were treated than to the technical competence of the dentist.

## 1. Introduction

Evaluating health services and monitoring the results of care are essential public health tools to transform various concepts and standards of measurement into key strategies. These strategies consequently contribute to the production of useful measures that assist in decision-making and improvements in services, schedules, and plans [1].

Thus, evaluations of patient satisfaction are considered an important part of the process of improving health services and have been increasingly used in recent years [1,2]. Patients’ perceptions of health services are influenced by their expectations of the quality of these services and the actual care received [2].

In 1988, Brazil established the Unified National Health System, the SUS (in Portuguese, *Sistema Único de Saúde*), to ensure that the population would have access to healthcare services, including oral health services. These services were organized on the levels of models or healthcare networks (primary, secondary, and tertiary); however, in oral health service, only primary care had been implemented at that time [3,4,5].

In 2004, based on public health data on the epidemiologic profile of the population, the Oral Health National Policy was created. This policy proposed a reorientation of the oral healthcare model to increase people’s access to oral health care and implemented the structure of secondary care, offering specialized services, including endodontics [3,6].

Among the measures implemented and financed by the Brazilian Ministry of Health, Dental Specialty Centers (DSCs; in Portuguese, *Centros de Especialidades Odontológicas*) were created. These centers are regionally distributed by the municipalities of the Brazilian states and are integrated into the local and regional planning process [3,7,8] and the healthcare network [5].

Beyond endodontic treatment, DSCs provide other specialized services such as periodontal treatments, minor oral surgery, oral diagnosis, and care for patients with special needs [7]. Unitary prosthesis is not offered, which can make it difficult to complete the rehabilitation of endodontically treated teeth. A patient must seek such services on his or her own behalf. These DSCs can contribute to the building of a healthcare network, defined as a set of articulated actions and health services, at increasingly complex levels with different technological densities to ensure the integrality of healthcare. This strategy improves health, reduces the incidence of disease, and contributes to reducing inequity [5]. Many countries in Latin America, such as Colombia, Brazil, Chile, Mexico, Uruguay, and Argentina, have promoted the development of these networks to provide a coordinated continuum of services to a defined population [9].

Advances in investments and planning in Brazilian public oral health have grown, targeting social inclusion and improved service. Service evaluations are thus necessary to plan and adopt strategies for targeting the Brazilian public health resources [10], specifically the service of DSCs, recently created in Brazil. Furthermore, evaluations by users of this system are also part of this process. Thus, this study evaluated patients’ perceptions of endodontic treatment performed as part of public health service in three cities in Minas Gerais, Brazil.

## 2. Materials and Methods

The study was conducted in three cities of Minas Gerais, Brazil, located close to the capital of the state in the central region (metropolitan area). These cities presented similar socioeconomic indicators and were selected for convenience because their secondary oral health services were well organized and structured.

This qualitative research methodology was based on semi-structured interviews and a field diary as an auxiliary technique to determine patients’ perceptions of endodontic treatment performed in the three selected cities. The participants were patients older than 18 years and had received endodontic treatment in a DSC in one of the three selected cities between March and April 2012.

This study was based on the quality of care framework proposed by Donabedian [11,12], in which quality is evaluated using structure, process, and outcome parameters. The focus of this study was the process [11] from the patient perspective.

A semi-structured interview format with a pre-determined set of questions was used. The questions reflected the patients’ ability to access and finish receiving endodontic services in their city and targeted the quality of these services (Table 1). A pretest was conducted by researcher José Leonardo Barbosa Melgaço-Costa with one patient who did not participate in the main study to test and suitably adjust the research instrument. 

The researcher spent three days in each city, and he interviewed individuals who volunteered and agreed to cooperate. All interviews were conducted by the same researcher José Leonardo Barbosa Melgaço-Costa in a quiet and private room after the patients’ endodontic treatments had been completed. Each interview lasted approximately 30 min. The researcher was not a clinician in any of the evaluated cities and had contact with the participants only at the time of the interview. After 10 interviews, we reached data saturation [13], and no more relevant new material could be obtained. All interviews were audiotaped and transcribed verbatim by José Leonardo Barbosa Melgaço-Costa.

Direct observations of public health services were noted in the field diary, and these observations consisted of facts and circumstances concerning the work process of the units and/or the interview or treatments and observations related to the problem under investigation [13]. Furthermore, notes were made in conversations with endodontic service professionals, including managers, dentists, and dental assistants. During these observations and after each session of treatment, the number of appointments and the type of technology employed were noted to complement the interviews. These notes were made at the end of each interview to avoid constraining patients.

The data were analyzed using content analysis [13]. This process involved performing several readings of the verbatim transcripts and field notes and listening to the audio recordings by José Leonardo Barbosa Melgaço-Costa, Renata Castro Martins, and Efigênia Ferreira Ferreira to extract expressions and words leading to the central themes. The researchers discussed disagreements until a consensus was reached.

This study was approved by the Human Research Ethics Committee at the *Universidade Federal de Minas Gerais* in Brazil (Protocol ETIC 0718.0.203.000-11), and all patients signed an informed consent form.

## 3. Results and Discussion

Ten patients aged 18 to 75 years were interviewed. Following a thorough study of the transcriptions by three researchers, the thematic content analysis indicated that the categories converged into two principal themes: access to service and quality of service.

### 3.1. Theme 1: Access to Service

The concept of access to health care has changed over time. It is related not only to geographical (availability) and financial (ability to pay) aspects but also to less tangible aspects, such as cultural, educational, and socioeconomic considerations, revealing a much more complex form [14]. A lack of access to oral healthcare is a problem that is not exclusive to Brazil [15]. The United Kingdom [16] and Canada [17] also witness severe inequalities in access to oral healthcare. Similarly, in South America, a study in Chile also reported inequality in the access to these services [18].

In this study, patients reported difficulties in accessing public health services, emphasizing the insufficient number of professionals to meet the high demand for endodontic treatment and the problems in referring from primary to secondary care:

“(…) in reality, they were slow to call because the demand is too great; only one center meets the demand of all units of the city, and demand is very high.”

“(…) if possible, referring patients to this location for basic care may increase the number of professionals in the field and perhaps (…) accelerate the service received at the DSC.”

The difficulties in accessing public health services has been observed in other studies [19,20], emphasizing that access to dental assistance plays an important role in patient satisfaction.

In our study, difficulties related to access were usually associated with geographic barriers and problems in referring patients from primary to secondary care. A previous study [21] showed that a lack of access is primarily due to insufficient system resources and an inappropriate allocation of resources between regions or places.

One patient cited the necessity of having more professionals in the area to improve people’s access to specialists and make it easier to meet the demand for endodontists. Furthermore, patients reported difficulties related to geographic location; for example, in one city, some patients requiring endodontists live in rural areas. The patients in these areas face challenges related to transportation. Additionally, only one team of health service professionals caters to these rural areas, and the dentist usually attends his practice only once per week in each district area (field diary by manager). This pattern demonstrates that the existence of a service does not mean that it is readily accessed [14].

For difficulties related to geographic location, O’Donnell [21] suggested improving transportation systems to make it easier to transport people to services or services to people. However, because Brazil is very large, this approach requires significantly more centers and specialists that are better distributed throughout the country to improve the balance in oral healthcare for the population as a whole [7,14].

Access to endodontic care was influenced by the time spent forwarding patients from a primary care unit to a DSC. Patients perceive this difficulty but cannot comprehend it. Patients’ perceptions of the waiting time between the referral from primary care and the receipt of endodontic treatment were influenced by their levels of pain. If they were in pain, participants invariably considered the wait too long; however, if they were not in pain, the wait was somewhat tolerable: “*I waited about 3 to 4 months. But it depends on the situation... If my tooth is aching (…) (the wait) will be a long time*”.

A long wait for treatment indicates a lack of integration between different points of care and the disarticulation of policies that regulate secondary care, which produces barriers to ensuring integrated care, making this process in the health care network incomplete [22].

Patients find individual solutions to the problems identified, and, in this study, luck and protection were highlighted. O’Donnell [21] showed that the difficulty in access occurs at all levels of care, including the lengthy waiting times to obtain a vacancy, which may be less if luck plays a role. The patients in the present study offered similar experiences, reporting “luck” as a criterion for service inclusion: “*I got lucky! Some people are delayed (in accessing the service), but for me, no. I got lucky (...) because there was a vacancy*”.

Patients also reported using the influence of politicians to obtain access to public health services [23]: “*To be honest (...) I called the secretary of X (a city official) and asked her if he could help me get the treatment (...). That's how I got it.*” This approach violates the principle of universal access to health services at all levels of care. Thus, despite advances in increasing access to and coverage of services, the distance between health practices and principles established by the SUS still remains, especially for minority groups [24].

A discussion of priorities in the DSC was also raised, but it contradicted the universality of the Brazilian health system. Some specific regional studies in Brazil concluded that the planning of public dental services has been marked by a redistributive tendency or pro-equity, adopting strategies and priority goals, such as the role of care users in the planning of health networks [25,26]. In terms of the difficulty of achieving full coverage of the population, the public health service establishes priorities, including the use of a protocol (age, prevalence, severity) in some locations. Patients understand that this is a good rule that they must follow: “*Sometimes (...) people’s dental problems aren’t so bad, (…) sometimes they only require prevention (preventive treatment) (...) (But) there are many people whose mouths (…) feel horrible (...); there are definitely people worse off than me (...). It’s very unfair*”.

### 3.2. Theme 2: Quality of Service

Patients’ reports about service quality were principally related to the pain and anxiety that they experienced, the time and number of sessions they needed to complete the endodontic treatment, their relationship with the team of secondary care professionals, and whether they would recommend the service and professionals to other patients.

Further studies of the quality of healthcare could help improve the functioning of services and comprehension of the criteria that patients use to rate service quality [1]. In this study, the absence of pain was defined as patients reporting no pain during endodontic therapy or between appointments. None of the patients reported having pain during the endodontic therapy or between sessions. The use of the criterion “does not feel pain” to evaluate care was consistent with existing findings in the scientific literature [27].

Although two endodontic treatment techniques were used in the DSCs studied (stainless steel hand instrumentation or nickel-titanium (NiTi) rotary instrumentation), according to the field diary, only one researcher perceived that patients who received treatment with rotary instruments had fewer appointments than those who received treatment with hand instruments. This fact was not noted by patients; they reported only that the sessions were relatively short, approximately one hour, with an average of approximately one week between each session. From the patients’ perspective, the number of sessions for endodontic treatment did not differ, irrespective of the technique used, as they did not realize the technical differences or time spent for each. The NiTi rotary instruments decreased the number of sessions needed to complete the endodontic treatment [28], and this useful fact can be used by public services to reduce the waiting time for endodontic treatment and to meet the demand for service, consequently increasing access [29,30].

Similarly, Donabedian [31] considered that the structural conditions of health services, which are the conditions that build and shape the universe of practices to be assessed (e.g., financial, human, physical, organizational resources), may favor the analysis of data in an evaluative process. Thus, it is recognized that a minimum structure is necessary but not sufficient to ensure high-quality care.

Aspects such as education, courtesy, and the ability to have a good relationship with the dentist were noted as important to service quality, even more important than the technical competence of the dentist. This result is consistent with one of the pillars of the evaluation of Donabedian care [12], which refers to the evaluation of the process, especially the interpersonal relationship between the health professional and the user. Patients emphasized the importance of attentiveness and good treatment: “*I like them (dentists); they have a light hand! For me, the main thing is to be (...) polite (...). If they don’t treat me well, I won’t return.*” Thus, these factors play key roles in how patients assess the quality of care [23,32,33]. These points were consistently observed across patient reports: Service quality and professional competence were associated more with the degree of humanity in their treatment and the information they received than with the actual treatment itself.

This study showed that patients often do not understand the nature of endodontic treatment. Many do not understand their treatment and must rely solely on the professional’s explanation and follow-up care (e.g., changing dressing, additional sessions), and they lack a clear understanding of the necessity of these instructions. This type of healthcare service, which is anchored in communication, may be the starting point for a more anthropological approach that contextualizes the structure of healthcare services, the socioeconomic circumstances of users, and the differences between the popular and medical models of health [32].

Some patients reported their satisfaction as follows: *“(It was) like private (treatment)”* or *“(...) Yes, you can trust (the treatment)! You don’t have to be afraid!”* Hence, although they were generally satisfied with the service quality, they also harbored negative preconceptions about the treatment provided by Brazil’s public health system. Other patients’ reports reinforce the notion that public service is the only option for many people: “*I liked it; it was very good, because if I had not been able to come here (…) and needed to pay, (…) I could not receive treatment because it is so expensive! When I was looking to pay for my own care, I found it was very expensive. I had no other, better option”.*

This observation indicates that some patients are not satisfied with or feel distrust toward public health services, believing that private health services are the model of quality [4,23]. Wallace and MacEntee [34] affirmed that this “fear” of the public health service is undoubtedly related to a general sense of vulnerability. Most of these opinions were likely anchored on a preconception built over time resulting from the absence of public health policies, especially oral health policies [3,4]. Furthermore, one study noted that individuals who are dark-skinned, those from larger households, those with a lower family income, those who live in small towns, and those who have a larger number of teeth that require treatment—in other words, the vulnerable population—are most likely to use public oral healthcare services [35].

It was observed that, although many patients evaluated the service positively, endodontic procedures were largely viewed negatively. Endodontic treatment is one of the most feared dental procedures and can sometimes result in tooth extraction because of fear and third-party reports of negative experiences [36]. Although many patients reported that the endodontists were careful, they also expressed fear of the treatment: “*People said that it (the treatment) hurt, but it was fine. I came expecting to feel pain, but I didn’t end up feeling any!*”

Regarding patients’ evaluations of the service as a whole, most commented on how well the treatment had met their needs. The patients were aware that the endodontic treatment was finished, but they had not yet undergone the necessary prosthetic treatment. In other words, their dental problems were partly resolved after the endodontic treatment. However, patients remained satisfied, which indicates that the reality of their experiences is profoundly influenced by their own expectations and assessment of the performance [2]. Conversely, because their root canals had been finished and they were no longer in pain, they found themselves generally satisfied with the endodontic service.

Although the SUS in Brazil is organized as a healthcare network to ensure the integrality of care [5], loss of teeth due to periodontal disease or endodontic infections is common, and the replacement of lost teeth with dental prostheses remains an inaccessible treatment for a large portion of the Brazilian population [7].

The ideal interface between primary and secondary care services is characterized by integrality, where every necessary treatment should be available and accessible at either the primary or secondary level. This treatment should be efficient and effective, ensuring appropriate referrals and adequate screening mechanisms. Furthermore, once treatment is completed, the reference counter should be assured [37].

In addition, criticisms of the treatment were related more to organizational issues than to technical issues: “*They called my house (...), but when I arrived here, it turns out I was on the other dentist’s schedule. So I my time was wasted!”* or *“(...) You should pay attention to what they do because the last time (…), they put (…) the X-ray of another person in my record.*” Thus, attention to the organization of resources, technical aspects of and changes in care, and the performance of workers is necessary to meet the basic principles of SUS and ensure equity [38]. 

Finally, when patients were asked if they would recommend the service to another person, they generally responded positively, stating that they recommend the service because of quality: “*I’ve already recommended (the service) to my friend (…). He started being evaluated. (...) I said that this place is cool.”* These recommendations could be considered a measure of quality and satisfaction, indicating increased confidence in the service [39].

User assessments can strengthen services by improving planning processes. Moreover, such assessments are a democratic form of expression and allow users of public health services to exercise their right as citizens, enabling them to participate in the development of health services, as recommended by SUS principles [1].

Qualitative studies, especially those with key informant interviews, are an important first step in determining critical issues and potential directions for further action, which are the strengths of this type of study, but they cannot produce data to be extrapolated to the population. The use of qualitative methods to investigate oral health policy can contribute to the identification of unique characteristics of health services that are specific to local and regional populations [40].

Information bias is one limitation of this study because patients may have reported a more favorable perception of a fear of losing the continuity of treatment achieved.

Finally, Sanchez and Ciconelli [14] highlighted that limited actions in the healthcare system prevent improvements in access to care and greater equity. Such improvements also depend on intersectional actions as well associal and economic policies that reduce income and education inequalities.

## 4. Conclusions

Access to endodontic treatment in secondary oral health care is a problem that was heavily emphasized by the participants in this study, and satisfaction with service quality was more related to the manner in which the patients were treated than to the technical competence of the dentist. Thus, patients’ qualitative assessments are a useful measure to improve health services. These assessments emphasize that patients are part of a wider process of evaluating services and should be used in conjunction with other assessment strategies.

## Figures and Tables

**Table 1:** Semi-structured interview questions.

Which tooth was treated at the DSC?
How many times did you come to the DSC to treat this tooth? What was done during each session?
What is your opinion of the treatment you received?
Approximately how long did you wait for endodontic treatment?
If you would recommend the endodontic treatment performed at this DSC to any person, who would you tell, and what specifically would you recommend about this treatment?
Did you finish the endodontic treatment of this tooth? Is the tooth completely treated (endodontic treatment and restoration of the tooth)?

## Abbreviations

The following abbreviations are used in this manuscript:

DSC	Dental Specialty Centers (in Portuguese, *Centros de Especialidades Odontológicas*)
NiTi	Nickel-Titanium
SUS	Unified National Health System (in Portuguese, *Sistema Único de Saúde*)
